# P118: Implementation of the World Health Organization multimodal hand hygiene improvement strategy in a pediatric Brazilian hospital

**DOI:** 10.1186/2047-2994-2-S1-P118

**Published:** 2013-06-20

**Authors:** AM Ribeiro, Francisca Luzilene N Della Guardia, Fernanda Calixto Martins, Virginia Maria Ramos Sampaio

**Affiliations:** 1 Control Infection, Hospital Infantil Albert Sabin, Fortaleza, Brazil

## Introduction

Hand hygiene is the cornerstone measure to prevent healthcare associated infection (HAI) and to ensure safe patient care. To improve compliance to hand hygiene (HH) and, decrease HAI, the Hospital Infantil Albert Sabin (HIAS) implemented the World Health Organization (WHO) Multimodal Hand Hygiene Improvement Strategy.

## Objectives

Assess the effectiveness of this strategy in terms of adherence to hand hygiene practice in a Pediatric hospital in Brazil’s northeast.

## Methods

The project was supported by the Brazilian government which formally signed an agreement in November 2007 to participate in the World Alliance for Patient Safety. The HIAS was selected by the National Health Surveillance Agency (ANVISA) Sentinel Network. The methodology followed the recommendations of the WHO and the original design was applied in three intensive care units (ICU) with 32 beds and 240 health workers in the period from 2008 to 2009. Compliance to hand hygiene was monitored by direct observation before and after intervention in the five moments indicated. The program Epi Info 6 was used to analyze data**.**

## Results

The rate of adherence to hand hygiene was 42% before intervention and 57.8% after intervention, an increase of 32% (p <0.001). The nursing technicians showed the lower adherence rate to HH before the intervention (35.5%), that increased to 56.1% after the intervention (p <0.001). Adherence to HH "before touching the patient" rate was 38.9% before and 59.7% after intervention (p <0.001). There was an increased use of alcohol gel from 8% to 62% in the actions of HH.

## Conclusion

It was found an increase in hand hygiene adherence by health workers in all three ICU during the implantation of the WHO multimodal strategy. This strategy will be implemented in all areas of the hospital care.

## Disclosure of interest

None declared

